# Upscaling human papillomavirus vaccination in high-income countries: impact assessment based on transmission model

**DOI:** 10.1186/1750-9378-9-4

**Published:** 2014-01-20

**Authors:** Iacopo Baussano, Joakim Dillner, Fulvio Lazzarato, Guglielmo Ronco, Silvia Franceschi

**Affiliations:** 1 International Agency for Research on Cancer, 150 cours Albert Thomas, 69372 Lyon cedex 08, France; 2 Karolinska Institutet, Nobels väg 12A, 17177 Stockholm, Sweden; 3 Department of Medical Sciences, Unit of Cancer Epidemiology, University of Turin, Via Santena 7, 10126 Turin, Italy; 4 Centre for Cancer Prevention, Via San Francesco da Paola 31, 10123 Turin, Italy

**Keywords:** Human papillomavirus, Vaccination, High-income, Mathematical model

## Abstract

**Background:**

The decrease in human papillomavirus (HPV) vaccine prices may allow upscale already started vaccination programmes but the advantages of different options are unclear.

**Methods:**

Using a mathematical model of HPV16 and 18 transmission and data on vaccination coverage from Italy, we compared 3 options to upscale an already started programme targeting 11-year old girls (coverage 65%): a) coverage improvement (from 65% to 90%); b) addition of 11-year-old boys (coverage 65%); or c) 1-year catch-up of older girls (coverage 50%).

**Results:**

The reduction of cervical HPV16/18 infection as compared to no vaccination (i.e. effectiveness against HPV16/18) increased from 76% to 98% with coverage improvement in girls and to 90% with the addition of boys. With higher coverage in girls, HPV16/18 infection cumulative probability by age 35 decreased from 25% to 8% with a 38% increase in vaccine number. The addition of boys decreased the cumulative probability to 18% with a 100% increase in the number of vaccinees. For any coverage in girls, the number of vaccinees to prevent 1 woman from being infected by HPV16/18 by age 35 was 1.5, whereas it was 2.7 for the addition of boys. Catch-up of older girls only moved forward the vaccination effectiveness by 2–5 years.

**Conclusions:**

Increasing vaccination coverage among girls is the most effective option for decreasing HPV16/18. If not achievable, vaccinating boys is justifiable if vaccine cost has at least halved, because this option would almost double the number of vaccinees.

## Introduction

The debate on the best target population of human papillomavirus (HPV) vaccination programmes in high-income countries has been renewed by the decision to vaccinate boys in Australia [1,2] and the similar recommendations from the United States [3] and by the relevance of non-cervical HPV-related cancers [4]. A key issue is whether an upscale of existing vaccination programmes should aim for increased vaccination of girls and young women or extend vaccination programme to boys [5]. The steady decrease in HPV vaccine prices makes an upscale attractive also for those high-income countries which initially targeted only one or few birth cohorts of girls or achieved low coverage.

The impact of vaccinating adolescent girls (and in some cases also boys) with or without catch-up of older girls has been explored using mathematical models [6-12]. The main aim of these studies was to estimate the incremental cost-effectiveness ratio of different vaccination options [7]. However, cost-effectiveness models are sensitive to the broad variations in the cost of HPV vaccines in different countries and depend on a variety of assumptions regarding incidence, incubation time and incidence rates of the various HPV-associated diseases.

In the present paper, we assess, as an example, different upscaling options in the vaccination programme that started in 2008 in Italy assuming that a fixed amount of resources has been allocated to HPV prevention. Our aim is to compare the effectiveness for HPV infection control of different options for an upscaled vaccination programme, in relation to the effectiveness of the existing baseline programme. We focused on viral endpoints (i.e. HPV16/18 cervical infections), which, though not clinically relevant *per se*, will be the earliest to be monitored. Three options were explored: a) coverage improvement of initially targeted girls; b) catch-up of a few additional female birth cohorts; and c) the addition of boys.

## Methods

The transmission dynamic model of HPV infection used in the present paper has been extensively described elsewhere [13]. Briefly, it is a population-based single-type model, which accounts for susceptible, HPV-infected and immune men and women. Each model’s output was fitted to the age-specific (25–29 to 55–59 age group) prevalence curves of cervical HPV16 and 18 infection that were observed in a large (94,370 women) population-based randomized controlled trial of cervical cancer screening (the New Technologies for Cervical Cancer, NTCC, trial) [14]. A total of 100,000 sets of parameter values were generated by independently sampling a uniform distribution for each parameter within a pre-specified range of values [15]. Each set of values was used to generate a model-based age-specific prevalence curve. Finally, the fit of each model’s output to the above-mentioned age-specific prevalence of HPV16 and 18 was assessed by calculating binomial log-likelihood. We selected the 100 model-generated curves that best fitted the observed data and among them we computed, for each parameter, the median and interquartile range (IQR) values as estimates of the most credible parameter values [13]. In order to account for the uncertainty of model-based estimates, the above-mentioned 100 best fitting curves for HPV16 and 18 were used to project the consequences of introducing different vaccination options.

**Figure 1:**
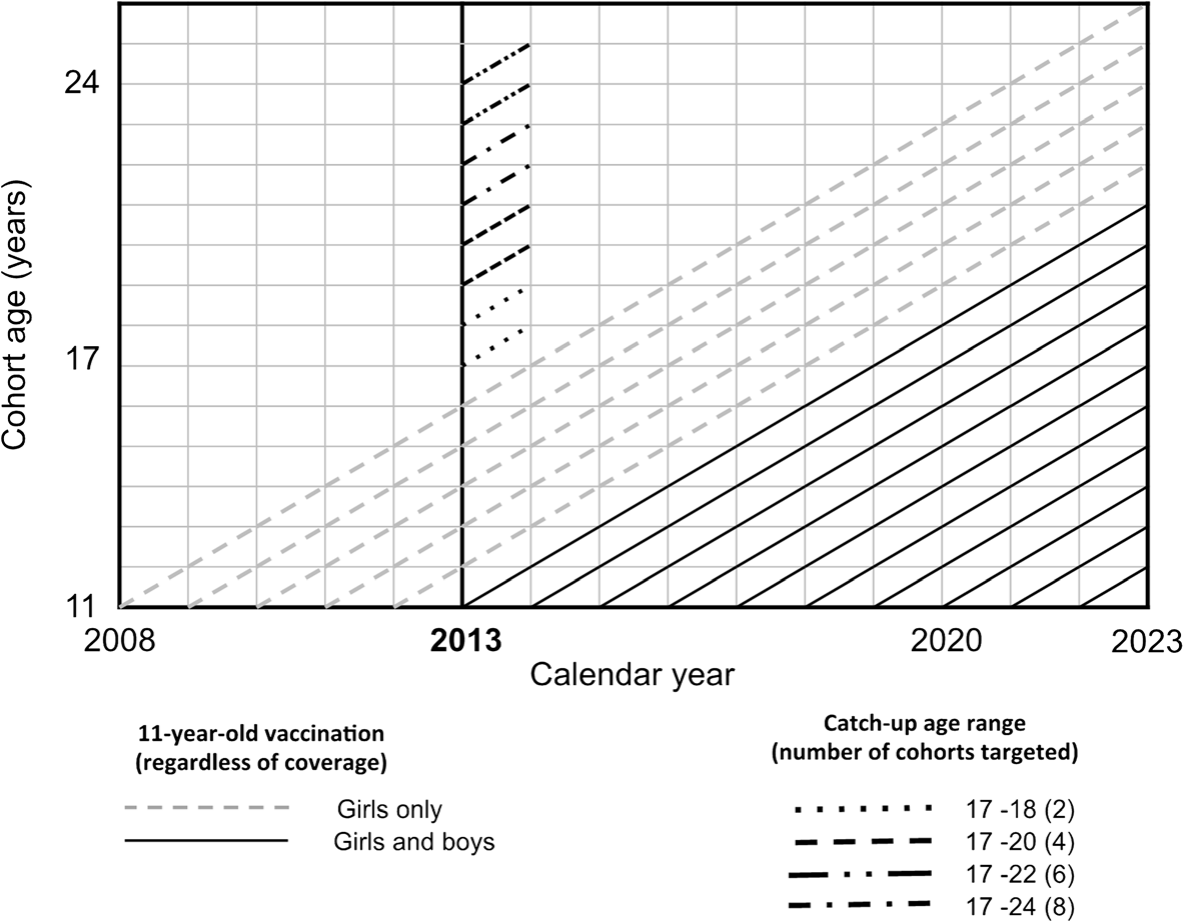
**Timing of the HPV vaccination programme as assumed for modelling purposes.** Legend: Baseline 11-year-old girl vaccination started in 2008; new options (catch-up of 17-to-24-year-old women; increased 11-year-old girls’ coverage; 11-year-old boys’ vaccination) started in year 2013. HPV: human papillomavirus.

HPV16 and 18 cervical infections were modelled separately as independent infections [16-18]. Clearance of infection was modelled assuming that it would be followed by natural type-specific immune protection in a fraction of women (i.e. 20% and 43%, for HPV16 and 18, respectively). The prevalence of HPV16 and 18 in the observed data was 2.4% and 0.7%, respectively, while 5% of HPV16 and/or HPV18 infected women were co-infected [13]. We eventually reported the impact of vaccination against the combination of the two types (HPV16/18). Vaccine efficacy against HPV16 and 18 infections among women was assumed to be 95% [19,20]. Vaccine efficacy among men was assumed to be 79% against HPV16 infection [21], and 95% against HPV18 infection [21]. The base case of an existing vaccination programme was derived from the Italian organized vaccination programme [22,23], i.e., initial vaccination of 11-year-old girls in 2008 (hereafter referred to as baseline vaccination) and a coverage of 65% [24] (Figure 1). We assessed the effectiveness of changes in the vaccination programme five years after its inception (2013) using three not mutually exclusive options: a) increasing 11-year-old girls’ coverage to 90% (the target initially set by Italian public health authorities); b) a 1-year catch-up campaign (national coverage assumed as 50%) that targets 17-to-24-year-old women; and c) vaccination of 11-year-old boys, with 65% coverage. As for sensitivity analyses, we repeated the projections increasing up to 65% coverage of catch-up and decreasing the coverage of 11-year-old boys down to 50%.

For each simulated upscale option, we present: 1) the effectiveness of vaccination as a function of calendar year after vaccination; and 2) the percent cumulative probability (CumProb) of HPV16/18 cervical infections by age 35 years according to birth cohort. Effectiveness was defined as the relative reduction in prevalence of HPV16/18 infections in women aged ≤35 years as compared to that in the absence of vaccination. Finally, we directly compared the different vaccination options for a limited number of birth-cohorts by calculating the number of subjects needed to vaccinate (NNV) in order to prevent a woman from being infected by HPV16/18 by age 35. The NNV was calculated assuming that birth cohorts were of the same size and included the same number of boys and girls. The corresponding number of vaccine doses may vary depending on the recommended vaccination schedule.

## Results

**Figure 2:**
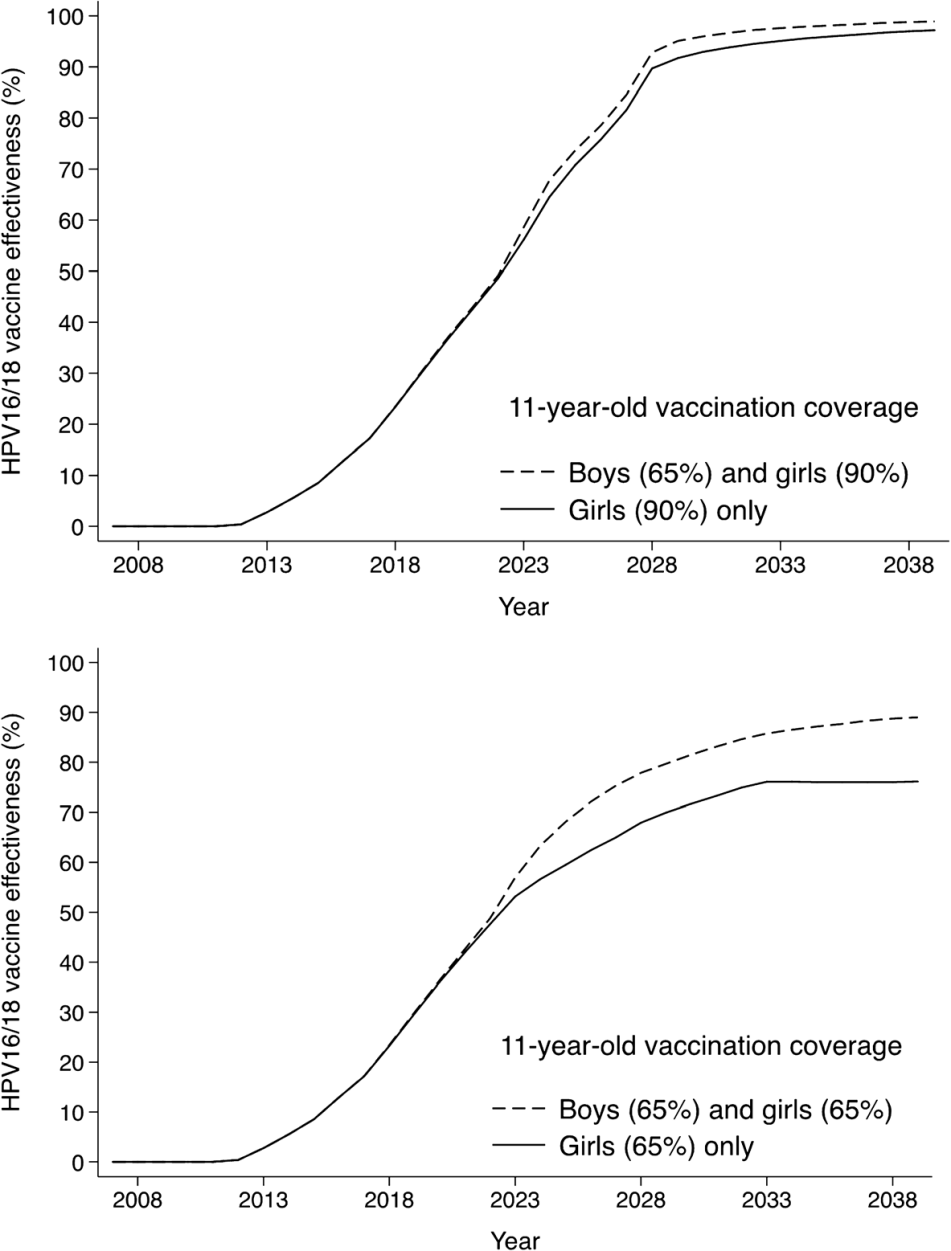
**Effectiveness (%) of vaccination against HPV16/18 infection, by coverage.** Legend: Effectiveness assessed among women ≤35 years, by 11-year-old girls’ coverage and addition of boys. HPV: human papillomavirus.

The improvement of vaccination coverage from 65% to 90% in 11-year-old girls increased the effectiveness against cervical HPV16/18 infections within 30 years since vaccination introduction from 76% to 98% (Figure 2). As a result of herd immunity (i.e. indirect protection of unvaccinated individuals) [25], effectiveness against HPV16/18 exceeded by 14 and 13 percentage points the one expected if vaccination had only protected vaccinated women (62% effectiveness at 65% coverage and 86% at 90% coverage).

The addition of 11-year-old boys to the vaccination of 11-year-old girls would raise maximal effectiveness to 90%. For both vaccinating boys and increasing coverage, the maximal effectiveness was 99% (Figure 2). The contribution of herd-immunity to vaccination effectiveness was 28 and 14 percentage points, respectively. Lower coverage of boys (i.e. 50%) would result in lower effectiveness.

**Figure 3:**
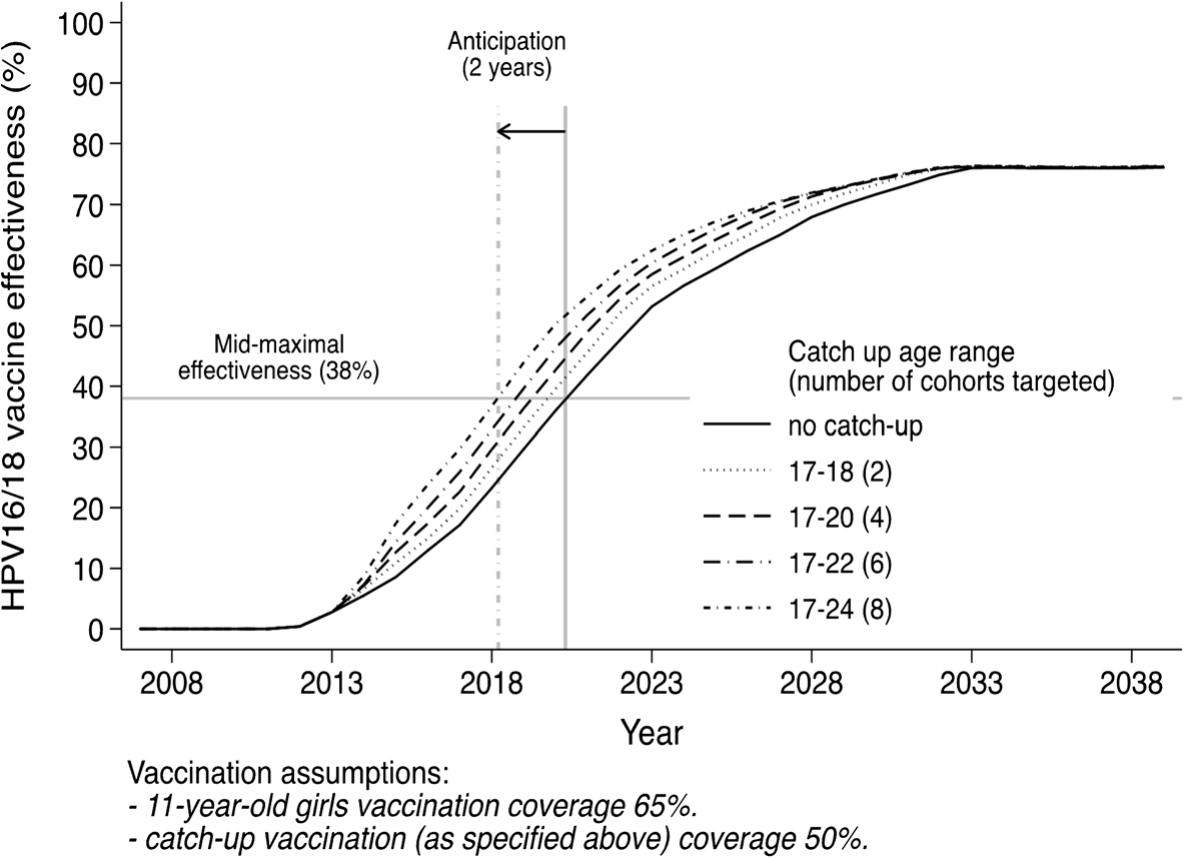
**Effectiveness (%) of vaccination against HPV16/18 infection, by catch-up.** Legend: Effectiveness assessed among women ≤35 years, by catch-up of 17-to-24 year-old women. HPV: human papillomavirus.

Catch-up of 17-to-24 year-old women did not affect the long-term effectiveness but it substantially moved it forward compared to baseline vaccination (Figure 3). The anticipation of effectiveness increased, according to a diminishing return pattern, as a function of the number of birth cohorts included in catch-up. For example, catch-up of 17-to-24 year-old young women moved forward mid-maximal effectiveness (38%, i.e., half of the effectiveness expected within 30 years as reported above) against HPV16/18 cervical infections by 2 years. Increasing catch-up coverage to 65% would anticipate effectiveness by about 5 years (data not shown). In no case did vaccination of adolescent boys produce a move forward of programme effectiveness, regardless of girls’ coverage.

Table 1 shows the median and IQR estimates of HPV16/18 CumProb by age 35, by birth cohort and upscale option. In absence of any HPV vaccination, the estimated CumProb was 67.5% (IQR: 56.9 – 75.1). Under the assumption of baseline vaccination of 11-year-old girls, starting with girls born in 1997 and with 65% coverage, CumProb estimates decreased down to 24.9% (IQR: 20.0 – 30.7) among girls belonging to the vaccinated birth cohorts. As the number of birth cohorts vaccinated increased, also the reduction in HPV16/18 CumProb by age 35 slightly increased.

**Table 1:** **Cumulative probability (%) of HPV16/18 infection by age 35**^**a**^**, by birth cohort and vaccination upscale option**

**Median cumulative probability [IQR] without vaccination**	**Year of birth**	**Girls vaccinated at age 11**	**Coverage of 11-year-old girls 65%, boys unvaccinated**^**b**^				**Coverage of 11-year–old girls**	
			**Number of cohorts of girls targeted by catch–up in 2013 (vaccination age range)**^**e**^				**90%**^**c**^	**65%**^**d**^
			**None**	**Two (17–18)**	**Four (17–20)**	**Eight (17–24)**	**Boys unvaccinated**^**c**^	**Boys vaccinated at age 11**^**d**^
	1993	No	64.9 [54.9 – 72.8]	63.8 [53.9 – 71.8]	45.9 [36.5 – 54.3]	45.3 [36.1 – 53.7]	64.5 [54.4 – 72.5]	64.4 [54.3 – 72.4]
	1996	No	43.8 [35.9 – 51.4]	31.7 [25.1 – 38.4]	31.0 [24.4 – 37.5]	30.7 [24.2 – 37.2]	42.9 [35.1 – 50.4]	42.8 [35.1 – 50.4]
67.5 [56.9 – 75.1]	1997	Yes	28.0 [22.6 – 34.1]	26.7 [21.6 – 32.7]	26.2 [21.2 – 32.0]	26.0 [20.9 – 31.8]	27.2 [21.8 – 33.0]	27.1 [21.8 – 32.9]
	1998	Yes	26.9 [21.9 – 32.9]	25.9 [21.1 – 31.8]	25.5 [20.7 – 31.5]	25.3 [20.5 – 31.3]	25.8 [20.7 – 31.5]	25.3 [20.6 – 31.0]
	2002	Yes	24.9 [20.0 – 30.7]	24.6 [19.7 – 30.6]	24.5 [19.5 – 30.4]	24.3 [19.4 – 30.2]	8.1 [6.0 – 10.2]	17.6 [14.1 – 21.5]

^a^We reported median estimates and corresponding interquartile range (IQR), according to year of birth cohort and catch-up strategy; ^b^Upscale option: 1-year catch–up; ^c^Upscale option: girls coverage improvement; ^d^Upscale option: addition of boys; ^e^Girls aged 12–16 not included due to 5-year delay in introducing catch–up (year 2013) after vaccination programme start (year 2008).

HPV: human papillomavirus.

Catch-up of older girls would lower the cumulative probability of HPV16/18 infections among women not initially targeted (Table 1). Targeting 17-to-18-year-old girls would reduce HPV16/18 CumProb by age 35 among 1996-born women to 31.7% (IQR: 25.1 – 38.4), but not among 1993-born women. However, the addition of more than four birth cohorts would not appreciably improve HPV16/18 CumProb (Table 1) because of the large fraction of young women sexually active above age 20.

In absence of catch-up, herd-immunity would also partially protect the last unvaccinated birth cohort (born in 1996), with an expected cumulative probability of HPV16/18 cervical infections of 43.8% (IQR: 35.9 – 51.4). The herd-immunity is expected to be lower among older birth cohorts as a consequence of decrease in sexual mixing with male partners of the vaccinated cohorts. For example, HPV16/18 CumProb among women born in 1993 would overlap the one expected in absence of vaccination (64.9%; IQR: 54.9 – 72.8).

The reduction of HPV16/18 CumProb by age 35 according to a) increase in 11-year-old girls’ coverage to 90%; or b) introduction of vaccination of 11-year-old boys in 2013 is shown in Table 1. Reduction was observed in cohorts born after 1997 in both options. The improvement of 11-year-old girls coverage reduced HPV16/18 CumProb to 8.1% (IQR: 6.0 – 10.2) in 2002-born women. The addition of boys without improving girls’ coverage decreased HPV16/18 CumProb to 17.6% (IQR: 14.1 – 21.5) in the same cohort.

**Figure 4:**
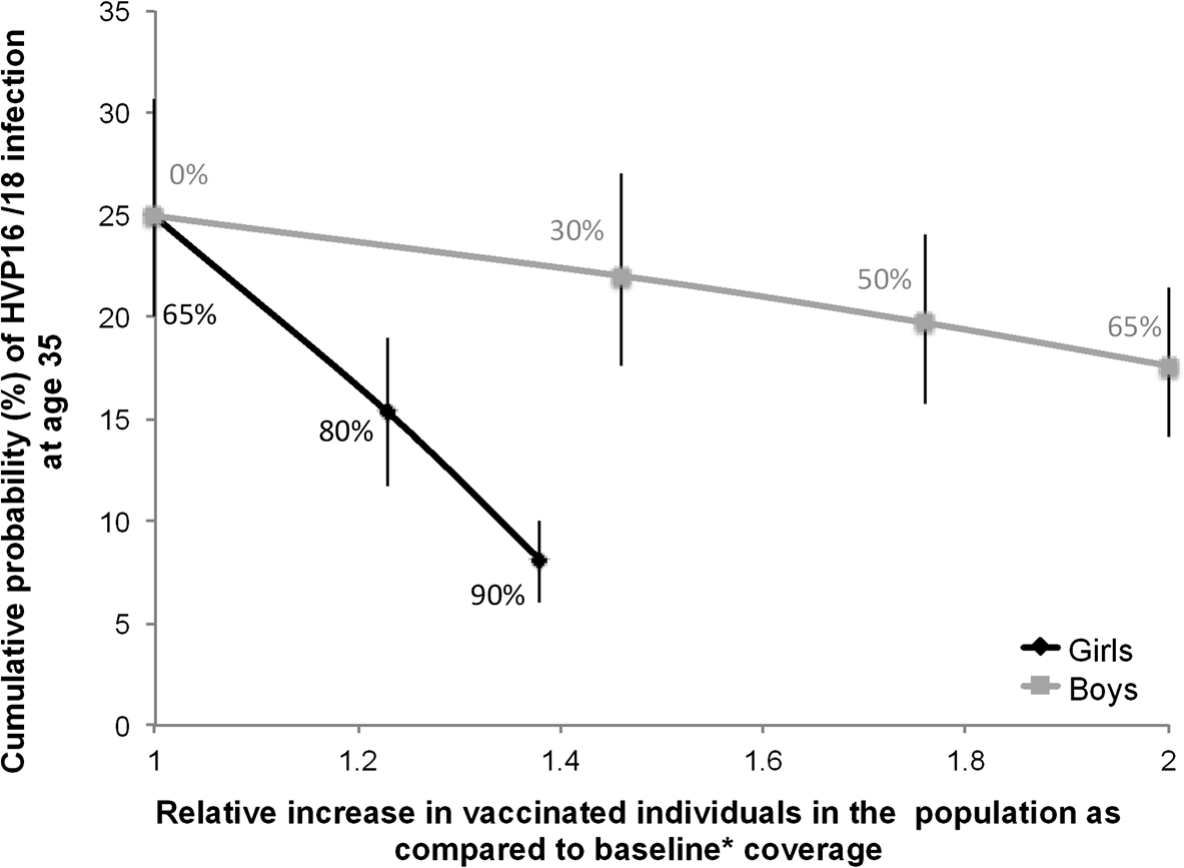
**Decrease in the cumulative probability (%) of HPV16/18 infection by age 35.** Legend: We report median and corresponding interquartile range (vertical bars), in the 2002-born women according to increase (%) in vaccinated individuals. Levels of vaccination coverage of 11-year-old girls and boys are also reported above or below the curves. Reference: baseline vaccination programme (11-year-old girls vaccination only: coverage 65%; no catch-up vaccination). HPV: human papillomavirus.

Figure 4 shows the decrease in HPV16/18 CumProb by age 35 among 2002-born women according to different upscale options. Horizontal axis shows the increase in vaccinated individuals relative to 65% coverage of 11-year-old girls only. The improvement of coverage of 11-year-old girls would reduce HPV16/18 CumProb from 25% to 8% and increase vaccinees by 38%. Vaccination of 11-year-old boys (without change in girl coverage) would lead to a reduction in HPV16/18 CumProb from 25% to 18% and increase vaccinees by 100%. Therefore, assuming a fixed amount of resources allocated to the vaccination programme, the improved coverage option would be acceptable if the vaccination cost *per capita* would be reduced by at least 28%, while the boys’ vaccination option would be acceptable if costs reduction would be at least 50%.

A direct comparison between vaccination options, restricted to the 10 first targeted birth cohorts, showed that the NNV was 1.5 under both the baseline and the improved girls’ coverage option. NNV was 2.7 if boys were also vaccinated (Table 2).

**Table 2:** Number of subjects needed to vaccinate (NNV) to prevent one woman from being infected by human papillomavirus (HPV) 16/18 by age 35

**Vaccination option**	**NNV**	**Ratio**
11-year-old girls only, coverage 65	1.5	Reference category
11-year-old girls only, coverage 90%	1.5	1.0
11-year-old girls and boys, coverage 65%	2.7	1.8

## Discussion

Our results show that improving vaccination coverage in adolescent girls is the most effective upscale option for decreasing the burden of HPV16/18 cervical infections in women. The addition of adolescent boys would approximately double NNV compared to girls’ vaccination but it can be considered useful and cost-neutral if the coverage of girls cannot be further improved and the cost per vaccination has at least halved.

A 1-year catch-up campaign of a few cohorts of older girls and younger women would move forward by about 3-to-5 years the impact of vaccination among targeted cohorts regardless of the vaccination strategy adopted for adolescent girls and boys. However, due to the higher probability of previous exposure to HPV16/18 infection, additional catch-up of older birth-cohorts would follow a diminishing return pattern. The catch-up of young women and improvement of vaccination coverage of 11-year-old adolescents would essentially benefit different birth-cohorts, i.e. those born in 2001 or earlier and after 2001, respectively.

Coverage of 90% of 11-year-old girls would increase the number of vaccinated adolescents by about 40% and decrease to 8% the HPV16/18 CumProb by age 35. The addition of 11-year-old boys with 65% coverage would double the PNN and decrease to 18% HVP16/18 CumProb by age 35 (Figure 4). While the improvement of girls’ coverage is clearly the most cost-effective upscale option (Table 2), the feasibility of achieving the improved coverage is uncertain. For example, a recent study from the US, where less than half of adolescent girls were fully vaccinated, suggested that fostering physician recommendation for vaccination may not be sufficient to increase HPV vaccine uptake in front of increasing families’ concerns about vaccine safety [26].

However, the cost of coverage improvement varies by setting, e.g., more expensive in on-demand programmes than in school- and/or by population-based invitations.

In Italy, for instance, vaccination against hepatitis B virus included catch-up of 12-year-old adolescents in *ad hoc* public clinics by active invitation between 1991 and 2003. Adolescent coverage reached 90% in most Italian regions [27]. Many conditions have, however, changed in the last decades including the current voluntariness of vaccinations, population’s attitude towards vaccines, and, in the case of HPV vaccination, the existence of screening as an alternative approach to cervical cancer prevention [28].

Our study shows that the different vaccination options also provide indirect protection against HPV16/18 to unvaccinated women. This indirect protection results from the decline in HPV transmission in sexual network that includes a high proportion of vaccinated women. Vaccinated women can indirectly protect the unvaccinated women by sharing sexual partners, while vaccinated boys directly prevent the transmission of HPV16/18 to unvaccinated women.

Our study has strengths and limitations. We focused on viral endpoints, although these are not clinically relevant outcomes, as they represent the earliest possible endpoints for monitoring vaccination programmes and the accuracy of model-based projections. We concentrated on HPV16/18 infections as information about natural history and vaccine cross-protection against other high-risk HPV types is limited. Restriction to virological endpoints up to age 35 minimizes the potential impact of different cervical screening practice on the interpretation of our findings [29]. Furthermore, we avoided the uncertainties inherent in estimating the cost and quality of life associated with the various HPV-associated diseases in men and women.

The generalizability of estimates based on dynamic model projection is largely dependent on the quality of assumptions of the parameters that govern natural history and sexual circulation of HPV infection. To test the validity of our sets of parameter values, we independently calibrated our Italian data-based model to Swedish data. Allowing for demographic and behavioural differences between the two countries and populations, the sets of parameters that regulate the natural history of HPV16 and 18 infections were shown to be consistent, suggesting that our projections are robust [13]. By contrast, our estimates, in particular for catch-up, are unlikely to satisfactorily apply to countries with significantly different patterns of sexual behaviours of adolescents and young women. Effectiveness of catch-up in our model was influenced by the Italian vaccination programme, in which HPV vaccination had been nearly restricted to 11-year-old girls for the first 5 years. Our findings, therefore, may be of particular interest for programmes with a similar initial set up. By contrast, the comparison of improving coverage of adolescent girls and/or vaccinating also boys is of interest to most high-income countries.

Our study only included estimates of vaccination benefit in women, although the reduction in incidence of HPV infection and HPV-associated cancers in men is an additional benefit of vaccination [30]. However, much fewer studies have addressed the natural history of HPV infections among men than women [31] and existing data are still insufficient to accurately calibrate a dynamic model that will allow reliable projections for males.

Due to the rapidly changing price of HPV vaccination, we chose not to include the monetary costs of purchasing and delivering HPV vaccines. However, the percent increase in vaccinated individuals in each vaccination option, as well as the NNV to prevent one woman HPV16 or 18 infected by age 35 should be easily applicable to country-specific vaccination costs in any given year and recommended number of vaccine doses [32]. NNV according to different options is especially useful as a guide to cost-effectiveness. If NNV doubles, as it is the case for boys, an upscale will be cost-neutral if vaccine cost had at least halved.

## Abbreviations

CumProb: Cumulative probability; HPV: Human papillomavirus; IQR: Interquartile range; NNV: Number needed to vaccinate; NTCC: New technologies for cervical cancer.

## Competing interests

IB, FL, GR, SF declare that they have no competing interests. JD has acted as consultant and received research grants from Merck and SPMSD.

## Authors’ contributions

IB, JD, GR and SF conceived and designed the study. IB and FL conceived, coded, and ran the transmission model. IB performed the analyses. GR and JD contributed to literature search, data collection and findings interpretation. IB and SF wrote the manuscript. All authors approved the final manuscript.
